# Cyclin D1 Overexpression Is Associated with Poor Clinicopathological Outcome and Survival in Oral Squamous Cell Carcinoma in Asian Populations: Insights from a Meta-Analysis

**DOI:** 10.1371/journal.pone.0093210

**Published:** 2014-03-27

**Authors:** Yanhui Zhao, Dedong Yu, Handong Li, Ping Nie, Yun Zhu, Shengwen Liu, Min Zhu, Bing Fang

**Affiliations:** 1 Department of Oral and Cranio-Maxillofacial Science, Shanghai Ninth People’s Hospital, College of Stomatology, Shanghai Jiao Tong University School of Medicine, Shanghai Key Laboratory of Stomatology, Shanghai, China; 2 Department of Medical Statistics, West China School of Public Health, Sichuan University, Chengdu, Sichuan, China; 3 Department of Oral & Maxillofacial-Head & Neck Oncology, Shanghai Ninth People’s Hospital, College of Stomatology, Shanghai Jiao Tong University School of Medicine, Shanghai Key Laboratory of Stomatology, Shanghai, China; Deutsches Krebsforschungszentrum, Germany

## Abstract

**Background:**

The clinicopathological significance of cyclin D1 overexpression and prognosis of oral squamous cell carcinoma has not been fully quantified. We performed a comprehensive meta-analysis for evaluation of cyclin D1 overexpression in oral squamous cell carcinoma to determine the strength of this association.

**Methods:**

Using both medical subheadings and free terms, we searched PubMed, Embase and the Institute for Scientific Information Web of Science for all eligible studies published before Nov. 2013. We retrieved 1674 citations, determining that 15 met the selection criteria. We used the odds ratio (OR) and hazard ratio (HR) as the common measures of association to quantitatively determine the correlation between cyclin D1 overexpression and outcomes of oral cancer. We performed a meta-analysis and heterogeneity, sensitivity, and subgroup analyses to clarify and validate the pooled results.

**Results:**

The pooled results provided compelling evidence that cyclin D1 overexpression was significantly correlated with increased tumor size (OR = 1.617, 95% confidence interval [CI] = 1.046–2.498, p = 0.031), lymphoid node metastasis (OR = 2.035, 95% CI = 1.572–2.635, p<0.001), tumor differentiation (OR = 1.976, 95% CI = 1.363–2.866, p<0.001), and advancement of clinical stages (OR = 1.516, 95% CI = 1.140–2.015, p = 0.004), and adversely influenced overall survival of OSCC patients (HR = 1.897, 95% CI = 1.577–2.282, p<0.001). The strength of association varied in different oral cavity subsites.

**Conclusion:**

Our findings indicated that cyclin D1 expression correlates with detrimental clinicopathological outcome and poor prognosis in oral squamous cell carcinoma. Our results may be useful in the management of oral cancer.

## Introduction

Oral squamous cell carcinoma (OSCC) is a common malignancy in the head and neck region, with significant incidence and mortality. In 2008, there were an estimated 263,900 newly diagnosed oral cancer patients and 128,000 deaths from oral cancer worldwide [1]. Despite recent advances in diagnosis and treatment, the 5-year overall survival (OS) rate remains at 50–60% [2].

In clinical practice, the conventional prognostic tool for cancers is the tumor-node-metastasis (TNM) system, in which lymph node metastasis is the most relevant [3]. However, there are some disadvantages to this system, namely difficulty in discriminating lymph node status in a timely and accurate manner, when using current physical examinations and imaging techniques. In addition, the biological phenotypes of tumors are often divergent despite identical staging, resulting in different clinical outcomes and response to the selected treatment [4]. Clarifying the correlation between biological characteristics or molecular biomarkers and the aggressiveness of oral cancer may provide be of significant benefit for predicting clinical outcomes and determining the optimal individualized therapy for each patient.

More recently, attention has focused particularly on a panel of molecular markers, which includes cell cycle regulators, as possible predictors of biological behaviors in oral cancer [5]. Cyclin D1 is a vital protein that has a widespread role in cell cycle regulations, providing control over G1 to S phase transition and governing cell proliferation rates [6], [7]. In the dynamic regulation over the cell cycle, Cyclin D1 exerts its functions by binding cyclin-dependent kinases (CDKs) subunit 4 and 6 to form a complex, which results in successive phosphorylation of the retinoblastoma (Rb) protein. On the other hand, this complex activates cyclin E-CDK 2 through sequestering the CDK inhibitors p21 and p27 [8]–[12]. The phophorylation of Rb results in its functional inactivation, and further leads to the release of E2F transcription factors, and proceeds to activate genes that are essential to initiate the DNA replication and accelerates cell proliferation [8]–[11]. These procedures will also in turn lead to the transcriptional activation of E2F-responsive genes that are essential to cyclin E and cyclin A synthesis, therefore further promote the phosphorylation of Rb through activating CDK2 [12]. Aberrant cyclin D1 expression, either by rearrangement, amplification or transcriptional up-regulation, contributes to the loss of normal cell cycle control and is associated with increased risk of tumorigenesis [4], [13]. In addition, cyclin D1 overexpression has a direct role in cooperating with other proto-oncogenes in neoplastic transformation in several systems [14]. Previously, it was extensively reported that cyclin D1 overexpression was as an important genetic event in a variety of head and neck cancers [15]–[17], including OSCC [4], [11], [18]. In oral cancer patients, immunohistochemical studies indicated a relation between certain prognostic factors and cyclin, including primary tumor size, location, nodal metastasis, tumor differentiation and clinical stage. However, the conclusions of such studies were not always in agreement. It is not known whether the heterogeneity originates from an actual difference or a lack of statistical power due to the relatively small sample size in an individual study.

Therefore, we performed a systematic review and meta-analysis of all eligible studies published published to date to gain deeper insight into the clinicopathological and prognostic significance of cyclin D1 in OSCC. We found a significant correlation between cyclin D1 overexpression and clinicopathological outcome and prognosis.

## Methods

### Search Strategy

We searched PubMed, Embase and the Institute of Scientific Information Web of Science for eligible studies published before November 2013. Searches were carried out using both medical subheadings and free terms. We used a combination of the following search string: (“oral” OR “mouth”) AND (“cancer” OR “carcinoma” OR “neoplasm” OR “tumor”) AND (“cyclin D1” OR “CCND1”) AND (“prognosis” OR “prognostic” OR “marker” OR “survival” OR “clinicopathological”). In addition, we manually screened the reference lists of included studies for further relevant studies. If the identified studies reported on overlapping populations, we selected the study that was published more recently or that contained more information.

### Selection Criteria

Two reviewers (Zhao Y. and Yu D.) screened the study selection process independently and in duplicate. Inter-reviewer agreement of the eligibility of the studies between reviewers was good, the kappa value was 0.9. Any disagreement was resolved by arbitration until consensus was achieved. Studies were eligible for inclusion if: (1) they were original articles published in either English or Chinese; (2) they focused on the association of cyclin D1 overexpression with high-risk clinicopathological factors and OSCC prognosis (3) they used immunohistochemistry (IHC) as the main method to examine the cyclin D1 expression in OSCC specimens. We restricted the included studies to those on Asian populations as previous studies have reported on these populations extensively. We excluded studies with no clinicopathological data. Studies on cutanous, verrucous and lip carcinoma as well as other types of carcinoma were also excluded.

### Data Extraction

All data for selected full-text articles were extracted by two independent reviewers (Zhao Y. and Yu D.) using standardized Excel 2007 worksheets (Microsoft, Redmond, WA). Discrepancies were resolved by discussions and referring to the contents of the articles. We extracted the basic study information (name of first author, year of publication, region or country in which study was conducted, size of study population), participant characteristics (recruitment period, sex and age distributions, treatment modality, duration of follow-up), IHC methodology (staining sites, cyclin D1 cut-off value) and clinicopathological parameters (tumor size, nodal metastasis, histological grade, clinical stage) from each study, and recorded the survival results of each study. From studies that reported hazard ratios (HR) in both univariate and multivariate models, we extracted the latter because these results were more convincing, as there had been adjustment for potential confounders.

### Statistical Analysis

We used the odds ratio (OR) as a common measure of association to determine the correlation between cyclin D1 expression and clinicopathological outcomes of oral cancer. The HR was used for quantitative evaluation of the impact of cyclin D1 expression on survival rate. Some studies did not include the point estimates and HR variance, therefore we used the data available in such studies and applied the method reported by Tierney et al. to determine the HR and its 95% confidence interval (CI) [19]. If a study reported only the survival curve, we extracted time-to-event data from the Kaplan–Meier curves of individual studies using Engauge Digitizer 4.1 software (free software downloaded from http://digitizer.sourceforge.net/).

For meta-analysis, the statistical significance of pooled estimates was determined using the Z-test. Heterogeneity across studies was checked by a chi-square based Q test and the Higgins I^2^ [20]. I^2^ represented the proportion of inter-study variability attributed to heterogeneity rather than systematic error, which ranges from 0% to 100% [21]. It was suggested that I^2^ values of 25%, 50% and 75% indicated low, moderate and high bias, respectively [21]. For a Q statistic p-value of >0.1, we used a fixed-effects model (the Mantel-Haenszel method [22]) to calculate the pooled estimates; otherwise a more conservative random-effects model (the DerSimonian–Laird method [23]) was used. However, in the rare events where incidence was <1%, we used the Peto one-step method instead [24]. This method tends to yield the least biased result and strongest statistical power, providing the best CI coverage and no substantial imbalance between case and control sizes [24]. In addition, we performed subgroup analysis to control for potential confounding factors as possible heterogeneity that might have distorted the results. Sensitivity analysis was performed using the leave-one-out method to test the reliability of the overall pooled results [25]. Funnel plot asymmetry was inspected visually to assess the possible effect of publication bias, which was confirmed by Egger’s linear regression [26].

All statistical tests in this meta-analysis were performed using Stata 11.1 software (Stata Corp, College Station, TX) with two-sided p values. A p-value <0.05 was considered statistically significant.

## Results

### Search Results

**Figure 1:**
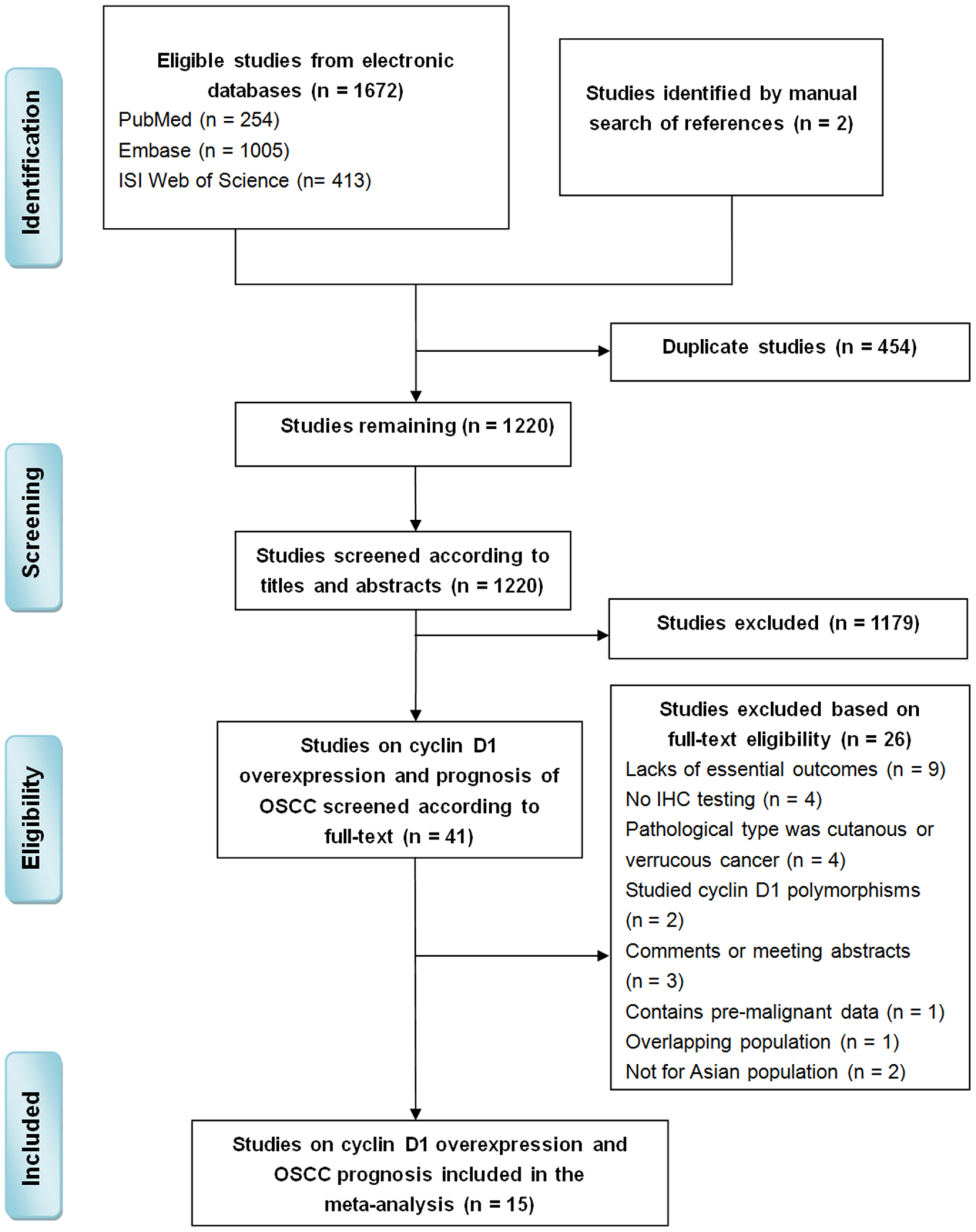
Studies selection flowchart.

Fig. 1 details the selection process. Our search strategy retrieved 1674 unique citations: 254 from PubMed, 1005 from Embase, 413 from the Institute for Scientific Information Web of Science, and two additional studies from the reference lists. After initial screening of the titles and abstracts, we excluded 1633 articles either because of duplication or they did not include the topics cyclin D1 overexpression and oral cancer. An eventual 41 articles underwent full-text evaluation. Upon further review, 26 articles were excluded nine had inadequate clinicopathological parameters for meta-analysis, four did not included IHC testing, four reported cutaneous or verrucous cancer, three were comments or meeting abstracts, two involved Caucasian populations, one contained pre-malignant data, two included genetic polymorphisms and one involved overlapping populations. Eventually, a total of 15 articles [8], [11], [13], [18], [27]–[37] were included based on the predefined criteria.

### Study Characteristics

Table 1 summarizes the characteristics of the included studies. All 15 articles included were of Asian origin; more specifically, six were from China [11], [13], [18], [24], [27], [37], 5 were from Japan [8], [29], [31], [33], [34] and 4 were from India [28], [30], [32], [35]. Altogether, the studies recruited a total of 1251 participants, with sample sizes ranging from 41 to 264 participants. All 15 studies used IHC methods for cyclin D1 staining. The cyclin D1 positive cut-off value varied between studies, ranging 5–50%. The most common selected cut-off was 10%. Regarding the clinicopathological factors, most of the studies reported prognostic factors of cyclin D1 expression referring to tumor size, nodal metastasis, histological grade and clinical stage. For survival analysis, eight studies reported OS [8], [11], [13], [30]–[33], [37], while only two studies investigated disease-free survival (DFS) as a potential outcome of cancer [11], [30].

**Table 1:** Characteristics of the included studies in this meta-analysis.

Study	Year	Recruitmentperiod	Country	Sampling	Tumorsite	Age(years)	Gender(M/F)	Duration offollow-up(months)	Treatment	Stainingpattern	Cut-offvalue	Cyclin D1Overexpression(%)
**Huang et al.**	2012	1999–2005	China/Taiwan	264	oral	49.33±11.01	264/0	168	surgery	nuclei andnon–nuclei	10	36.7
**Xing et al.**	2011	2005–2009	China	50	oral	20–7649.5	35/15	NA	NA	nuclei andcytoplasma	25	80
**Das et al.**	2011	2001–2006	India	45	oral	53.2±12.2	36/9	NA	surgery	nuclei	50	66.6
**Yun et al.**	2010	2004–2006	Japan	50	tongue	22–82	31/19	12–60(median 40)	surgery	nuclei	10	58
**Mishra et al.**	2009	NA	India	51	oral	NA	31/20	NA	NA	nuclei	NA	31.1
**Shah et al.**	2009	2000–2003	India	135	Buccal andtongue	28–75	101/34	>24	surgery and radio-,chemotherapy	nuclei andcytoplasma	10	43
**Wang et al.**	2006	2000–2003	China	62	tongue	25–86	40/22	NA	surgery	nuclei	10	66
**Shiraki et al.**	2005	1986–1998	Japan	140	oral	26–85(mean 59)	98/42	5–134(median 66)	surgery	NA	10	39
**Miyamoto** **et al.**	2003	1999–2001	Japan	41	oral	21–89(mean 58.4)	26/15	7.7–39.3(median 25.4)	surgery	NA	10	65.9
**Zhu et al.**	2003	1990–1999	China	50	oral	31–80(mean 60)	36/14	120	surgery	nuclei	10	52
**Vora et al.**	2003	1986–1990	India	84	tongue	NA	77/7	60	surgery (main),radiotherapy	nuclei	10	62
**Goto et al.**	2002	1981–1998	Japan	41	tongue	22–82(mean 59.6)	24/17	2–133(mean 36.3)	NA	nuclei	33	65.9
**Vicente et al.**	2002	1990–1999	Spain	35	oral	27–85(mean 56.6)	30/5	6–107(mean 68)	surgery,radiotherapy (37%)	nuclei	50	17.1
**Mineta et al.**	2000	1977–1995	Japan	94	tongue	16–89(mean 58)	68/26	>60	NA	nuclei	50	19
**Lam et al.**	2000	1988–1996	China/Hongkong	56	oral	37–85(mean 64)	45/11	>60	radiotherapy	nuclei	5	63
**Kuo et al.**	1999	1991–1995	China/Taiwan	88	oral	NA	76/12	>60	surgery,radiotherapy	NA	50	44.3
**Bova et al.**	1999	NA	Australia	148	tongue	NA	104/44	1–186(mean 57)	surgery,radiotherapy	nuclei	10	68

M/F: Male/Female; NA: not available.

### Quantitative Synthesis

**Figure 2:**
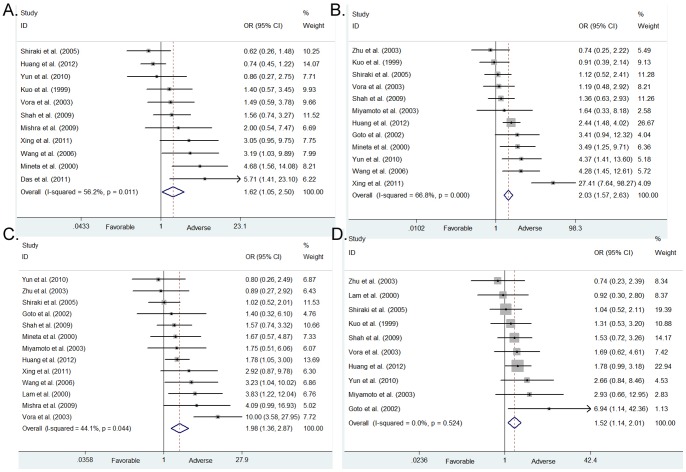
Forest plots of association between cyclin D1 overexpression with poor clinicopathological outcome in OSCC. (A). Tumor size, random-effects model; (B). Nodal metastasis, Peto one-step model; C. Histological grade, random-effects model; D. Clinical stage, fixed-effects model.

Eleven studies investigated the association between cyclin D1 expression and primary tumor size, which involves 1063 participants. Cyclin D1 overexpression was more prevalent in larger tumors (T3, T4) than in smaller tumors (T1, T2), and with OR = 1.617 (95% CI = 1.046–2.498) (Table 2 and Fig. 2). Some subgroup analysis maintained a significant association, more specifically, in the Indian populations (OR = 1.877, 95% CI = 1.148–3.068) and cut-off value >10% (OR = 2.752, 95% CI = 1.600–4.731) (Table 2). When we stratified the pooled estimates according to tumor site, there was a positive association between cyclin D1 expression and increased tumor size in tongue SCC (OR = 2.032, 95% CI = 1.200–3.441) rather than with a mixed tumor site in the oral cavity (OR = 1.431, 95% CI = 0.758–2.701) (Table 2).

**Table 2:** Meta-analysis results of association between cyclin D1 overexpression and clinicopathological outcomes in OSCC.

Outcomes	Variables	Subgroups	Study N.	Samples	Stat.	Test of association					Test of heterogeneity		
						OR	95% CI		Z	p_Z_	Het x^2^	p_het_	I^2^ (%)
**Primary tumor**	Total	Total	11	1063	R	1.617	1.046	2.498	2.16	0.031	22.85	0.011	56.2
	Country	China	4	464	R	1.579	0.735	3.392	1.17	0.241	8.99	0.029	66.6
		India	3	315	F	1.877	1.148	3.068	2.51	0.012	2.93	0.403	0.0
		Japan	3	284	R	1.323	0.385	4.544	0.44	0.657	8.44	0.015	76.3
	Cut-off	10%	6	735	F	1.028	0.751	1.407	0.17	0.861	8.74	0.120	42.8
		>10%	4	277	F	2.752	1.600	4.731	3.66	<0.001	4.14	0.247	27.5
	Tumor	mixed	6	638	R	1.431	0.758	2.701	1.11	0.268	13.44	0.020	62.8
		tongue	4	290	F	2.032	1.200	3.441	2.64	0.008	5.36	0.147	44.1
**Nodal metastasis**	Total	total	12	1099	P	2.035	1.572	2.635	5.39	0.000	33.16	<0.001	66.8
	Country	China	5	514	P	2.325	1.620	3.337	4.58	0.000	24.38	<0.001	83.6
		India	2	219	F	1.282	0.716	2.296	0.84	0.403	0.05	0.828	0.0
		Japan	5	366	F	2.215	1.361	3.604	3.20	0.001	6.12	0.190	34.7
	Cut-off	10%	8	826	R	1.754	1.131	2.719	2.51	0.012	12.10	0.097	42.2
		>10%	4	273	P	2.954	1.737	5.026	4.00	<0.001	19.12	<0.001	84.3
	Tumor	mixed	6	633	P	1.878	1.343	2.627	3.68	<0.001	25.26	<0.001	80.2
		tongue	5	331	F	2.915	1.761	4.826	4.16	<0.001	5.78	0.216	30.8
**Histological grade**	Total	total	13	1118	R	1.976	1.363	2.866	3.59	<0.001	21.48	0.044	44.1
	Country	China	6	532	F	1.841	1.279	2.649	3.29	0.001	6.61	0.251	24.4
		India	3	270	R	3.841	1.131	13.039	2.16	0.031	8.31	0.016	75.9
		Japan	4	316	F	1.271	0.777	2.078	0.96	0.339	0.92	0.821	0.0
	Cut-off	10%	8	826	R	1.781	1.076	2.947	2.25	0.025	17.74	0.013	60.5
		>10%	3	185	F	1.953	0.974	3.916	1.89	0.059	0.71	0.702	0.0
	Tumor	mixed	8	693	F	1.707	1.242	2.347	3.29	0.001	7.59	0.37	7.8
		tongue	4	290	R	2.600	0.894	7.564	1.75	0.079	11.53	0.009	74
**Clinical stage**	Total	Total	10	949	F	1.516	1.140	2.015	2.87	0.004	8.10	0.524	0.0
	Country	China	4	458	F	1.363	0.903	2.058	1.47	0.140	2.32	0.509	0.0
		Japan	5	356	F	1.728	1.091	2.737	2.33	0.020	5.25	0.262	23.9
	Cut-off	10%	7	764	F	1.530	1.112	2.103	2.62	0.009	4.51	0.609	0.0
		>10%	2	129	F	1.837	0.840	4.019	1.52	0.128	2.64	0.104	62.1
	Tumor	mixed	6	639	F	1.339	0.949	1.889	1.66	0.096	3.87	0.568	0.0
		tongue	3	175	F	2.482	1.250	4.930	2.60	0.009	1.82	0.403	0.0

N.: Number; Stat.: Statistic models; R: random-effects model; F: fixed-effects model; P: Peto one-step method; OR: odds ratio; 95% CI: 95% confidence intervals; p_ Z_: p value of statistic Z; p_ Het_: p value of heterogeneity chi-squared; p_ Z_ <0.05 was regarded as significant; p_Het_ <0.1 was regarded as significant.

We also summarized information associating cyclin D1 overexpression with other clinicopathological parameters as the topic of interest reported in the included studies, including nodal metastasis, histological grade and clinical stage (Table 2 and Fig. 2). The overall estimates indicated that cyclin D1 overexpression was significantly associated with increased risk of nodal metastasis (N1, 2, 3 versus N0) in oral cancer patients (OR = 2.035, 95% CI = 1.572–2.635). Such associations were also found for tumor histological grade (OR = 1.976, 95% CI = 1.363–2.866) and clinical stage (OR = 1.516, 95% CI = 1.140–2.015). When stratified according to tumor site, the pooled estimates changed significantly. The association with tongue SCC was more obvious than that in the oral cavity (nodal metastasis, OR = 2.915 versus 1.878; histological grade, OR = 2.600 versus 1.707; clinical stage, OR = 2.482 versus 1.339, respectively) (Table 2). In addition, we stratified the cut-off value, an important source of heterogeneity at 10%, because most of the studies used this criterion to denote cyclin D1 overexpression. Further, the positive rate of keratinocytes is <10% in normal oral mucosal epithelial [38]. The pooled estimates in the clinicopathological data were altered substantially following stratification of the cut-off value (Table 2).

The results associating cyclin D1 overexpression with clinical stage did not have potential heterogeneity (p_het_ >0.1), while there was some heterogeneity among studies following analysis of other clinicopathological features, with I^2^ ranging from 44.1 to 66.8% (Table 2).

**Figure 3:**
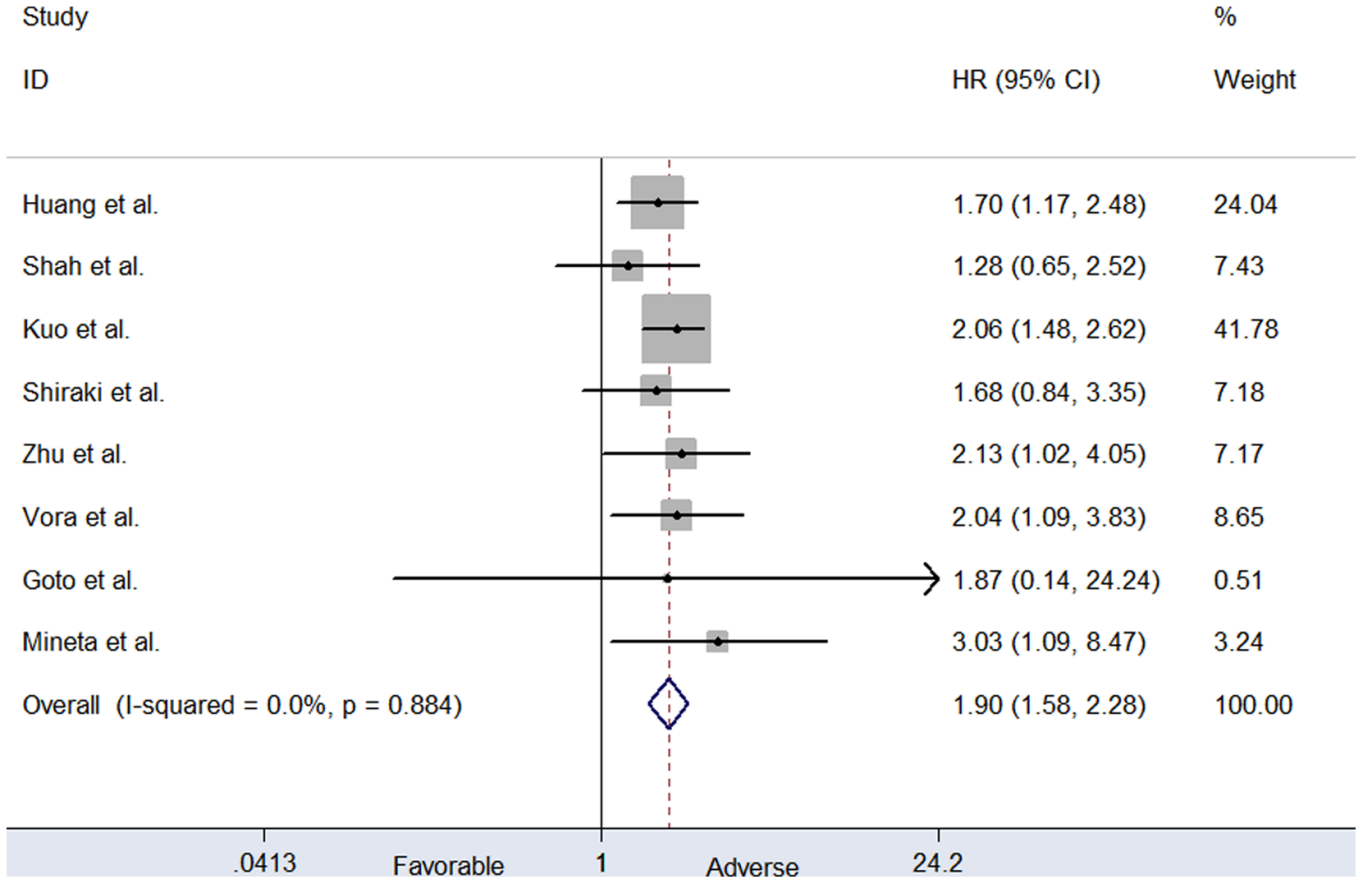
Forest plots of association between cyclin D1 overexpression with poor OS in OSCC.

To investigate whether cyclin D1 overexpression was a prognosis factor in oral cancer patients, we meta-analyzed the HR data extracted from individual studies or derived using the calculations described in the. Most of the studies indicated a stronger link between cyclin D1 overexpression and poor survival. For OS, mortality was higher in cyclin D1-positive groups than in cyclin D1–negative groups (HR = 1.897, 95% CI 1.577–2.282, p<0.001), with no potential heterogeneity across studies (p_het_ = 0.884, I^2^ = 0.0%) (Fig. 3). Significant association was also found for DFS (HR = 1.421, 95% CI 1.038–1.947, p = 0.028). There was no heterogeneity among studies in the pooled analysis (p_het_ = 0.403, I^2^ = 0.0%).

### Sensitivity Analysis

The overall pooled estimates of the relation of cyclin D1 expression to clinicopathological and prognostic outcomes were not substantially altered following the exclusion of any individual study, indicating the reliability of our results.

### Publication Bias Analysis

**Figure 4:**
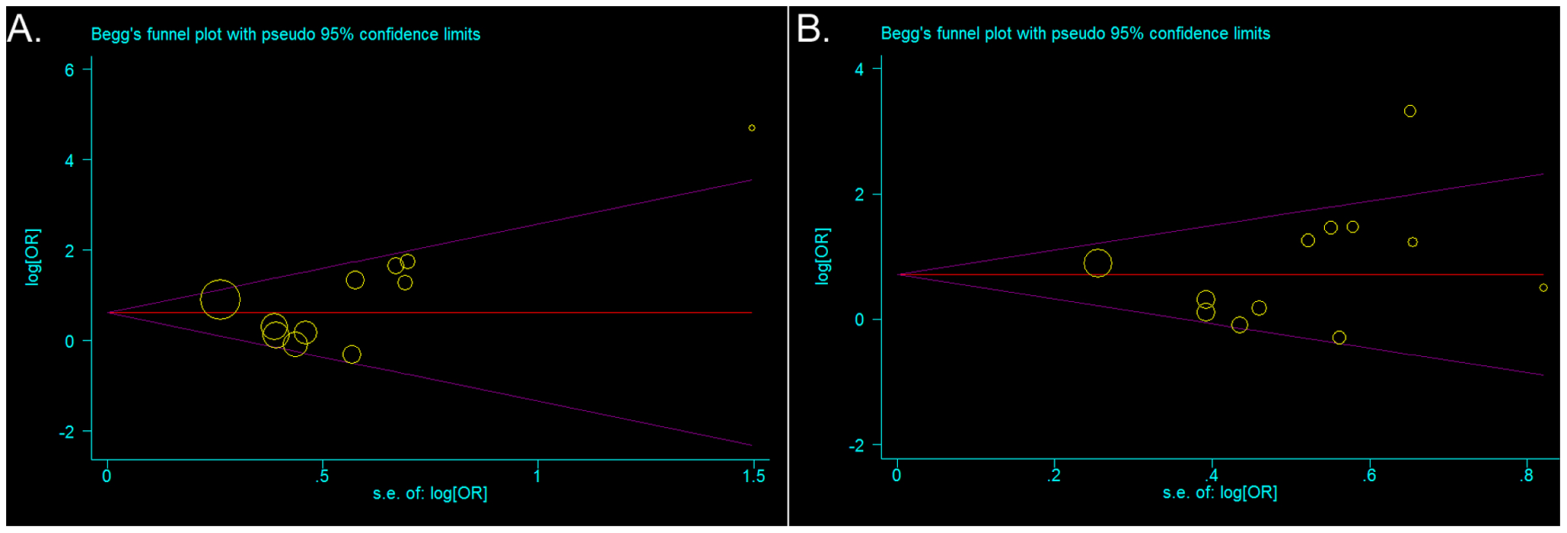
Begg’s funnel plots for publication bias in cyclin D1 overexpression and clinicopathological outcome in OSCC. Each point represents a separate study for the indicated estimate; the area of each circle represents the sample size. s.e: standard error; Horizontal line: effect size. (A). Funnel plots of publications for the association between cyclin D1 overexpression and nodal metastasis, random-effects model. B. Funnel plots of publications for the association between cyclin D1 overexpression and histological grade, Peto one-step model.

Analysis of clinicopathological features and survival data determined no obvious asymmetry in the funnel plots for publication bias (p>0.05) (Fig. 4). The more sensitive Egger’s regression test confirmed these results, demonstrating that our pooled data contained no potential publication bias.

## Discussion

Based on 1251 Asian OSCC patients from 15 studies, we explored the relation of cyclin D1 expression to clinicopathological features and survival in OSCC. Our pooled results are compelling evidence of a significant correlation between cyclin D1 overexpression and increased tumor size, tumor differentiation, lymphoid nodes metastasis, and advancement of clinical stage, which may adversely influence survival in OSCC.

Our findings are in agreement with a recent meta-analysis of esophageal carcinoma that combined the clinicopathological data of 2041 patients [39]. Various studies on head and neck SCC (HNSCC) involving large sample sizes also arrived at similar conclusions: Hanken et al. [16] evaluated the cyclin D1 expression status in 546 HNSCC patients finding a significant association cyclin D1 expression OS in oral subsites (p<0.001). Rasamny et al. [40], studied 222 samples of upper aerodigestive HNSCC, and reported that strong positive cyclin D1 expression was related to remarkable reduction of overall and disease-specific survival (OS, p = 0.003, disease-specific survival, p = 0.039 ). Together with these studies, our results suggest that cyclin D1 overexpression is related to local invasiveness and aggressive behavior of SCC, especially in the oral cavity.

In the subgroup meta-analysis, the strength of association of cyclin D1 expression in tongue SCC was stronger than in mixed tumor sites of the oral cavity. There may be two reasons for this difference: First, compared to other subsites in the oral cavity, tongue SCC is characterized by a more aggressive biological phenotype, with a high-degree of cervical lymph node spread that might be reflected at molecular level, such as with cyclin D1 expression. Second, cyclin D1 may be differentially expressed in various anatomical sites in the oral cavity [18], [41], [42]. Regarding ethnicity, two studies previously reported the clinicopathological significance of cyclin D1 overexpression in Caucasian populations. Vicente et al. [4] performed an IHC study in 35 Spanish OSCC patients, finding that cyclin D1 overexpression was related with 2.6 times greater nodal metastasis. Bova et al. [43] investigated 148 Australians with tongue cancer, reporting that cyclin D1 overexpression was associated with higher lymph node stage (approximately 3.43 times higher, p = 0.014), and lower DFS (p = 0.06) and OS (p = 0.01). Compared with our meta-analysis of Asian populations, these disparities may exclude racial difference in the association between cyclin D1 expression and oral cancer development. AS there were only two small studies on Caucasian populations [4], [43], it is possible that this observation has lower statistical power and increased sampling error. Therefore, future studies addressing cyclin D1 expression and OSCC in Caucasian populations are warranted to verify this association.

Certain heterogeneities were observed in our pooled analysis of the clinicopathological data. Heterogeneity across studies can likely be attributed to different IHC methodology, including the primary antibody used, antibody dilutions, and the scoring system applied. Asymmetric labeling of cyclin D1 expression in different parts of a specimen may also contribute to heterogeneity [7]. Moreover, disparity in the criteria for cyclin D1 expression can lead to potential bias among studies, as indicated in our review, with a variance of 5–50%. Unfortunately, we could not clarify the source of heterogeneity thoroughly through subgroup stratification and sensitivity analysis.

Although our systematic review was robust in identifying a correlation between cyclin D1 expression and poor clinical outcome in OSCC, this study should be interpreted in view of its limitations. First, this review was restricted to studies published in English and Chinese, which may have caused a potential bias. However, studies in other languages were not always available to us. Second, heterogeneity was not eliminated entirely although we conduct subgroup and sensitivity, which may have distorted the pooled results. Third, due to the lack of original data in the included studies, it was not possible to evaluate the combined effect of and interaction between cyclin D1 and other molecular markers on clinical outcomes. As the carcinogenesis of oral cancer involves a multi-hit process [32], multiple molecular tests might provide more detailed information on the prognosis and treatment of this cancer. Fourth, only a few studies reported the HRs and its 95% CIs. Although we attempted to obtain more information using recommended methods [19], information on HRs remained limited. Thus, we could not analyze the pooled HR either by data type (e.g. data from univariate or multivariate model) or subgroup analysis (e.g. ethnicity, treatment modality, cut-off value, age, sex).

Despite these limitations, our findings may have important clinical implications. Clinicians would find our conclusions helpful, as there is currently no molecular tool for evaluating the development and progression in oral cancer. As a vital cell cycle regulator, Cyclin D1 could be considered a valuable prognostic tool in OSCC treatment, when combined with conventional TNM staging. Much caution is advised when managing aberrant cyclin D1 expression in OSCC, as it indicates a high risk of regional metastasis and recurrence following surgical treatment. Such patients, however, may undergo a more thorough treatment modality and be followed more closely in clinical practice. Moreover, as preoperative evaluation of cervical metastasis lacks accuracy, we concur with the opinion that IHC analysis of cyclin D1 expression in diagnostic biopsy samples could be deemed a routine to select patients who can be probably treated with elective neck dissection [3], [4].

The current study also highlights the need for future research in this area. Studies associating cyclin D1 expression and oral cancer prognosis are warranted to better ensure ample sample size with varying in the study population, better control for potential confounders (e.g., using the same IHC methodology and cut-off value) and to take into account the interaction between cyclin D1 expression and environmental factors or personal habits (e.g., cigarette smoking, alcohol abuse, areca quid chewing). Such studies may have many advantages over the current study which mined small- samples individual studies. In addition, as the biological behavior of SCC in different oral cavity subsites may vary, more attention should be focused on investigating cyclin D1 expression and prognosis in individual oral cavity subsites. The prognostic value of cyclin D1, either alone or combined with alternative molecular markers, would be validated in clinical practice.

## Conclusion

Our meta-analysis indicates that cyclin D1 overexpression correlates with poor clinicopathlogical outcome and prognosis in OSCC. The results obtained may aid the management of oral cancer patients.

## Supporting Information

Checklist S1 PRISMA Checklist.
(DOCX)
